# Impacts of insect frass and cadavers on soil surface litter decomposition along a tropical forest temperature gradient

**DOI:** 10.1002/ece3.9322

**Published:** 2022-09-21

**Authors:** Bernice C. Hwang, Christian P. Giardina, Creighton M. Litton, Kainana S. Francisco, Cody Pacheco, Naneaikealaula Thomas, Tyler Uehara, Daniel B. Metcalfe

**Affiliations:** ^1^ Department of Physical Geography and Ecosystem Science Lund University Lund Sweden; ^2^ Pacific Southwest Research Station, USDA Forest Service Institute of Pacific Islands Forestry Hilo Hawaii USA; ^3^ Department of Natural Resources and Environmental Management University of Hawai‘i at Mānoa Honolulu Hawaii USA; ^4^ Department of Ecology and Environmental Science Umeå University Umeå Sweden

**Keywords:** insect herbivory, nitrogen mineralization, nutrient cycling, *Q*_10_

## Abstract

Insect herbivores play important roles in shaping many ecosystem processes, but how climate change will alter the effects of insect herbivory are poorly understood. To address this knowledge gap, we quantified for the first time how insect frass and cadavers affected leaf litter decomposition rates and nutrient release along a highly constrained 4.3°C mean annual temperature (MAT) gradient in a Hawaiian montane tropical wet forest. We constructed litterbags of standardized locally sourced leaf litter, with some amended with insect frass + cadavers to produce treatments designed to simulate ambient (Control = no amendment), moderate (Amended‐Low = 2 × Control level), or severe (Amended‐High = 11 × Control level) insect outbreak events. Multiple sets of these litterbags were deployed across the MAT gradient, with individual litterbags collected periodically over one year to assess how rising MAT altered the effects of insect deposits on litter decomposition rates and nitrogen (N) release. Increased MAT and insect inputs additively increased litter decomposition rates and N immobilization rates, with effects being stronger for Amended‐High litterbags. However, the apparent temperature sensitivity (*Q*_10_) of litter decomposition was not clearly affected by amendments. The effects of adding insect deposits in this study operated differently than the slower litter decomposition and greater N mobilization rates often observed in experiments which use chemical fertilizers (e.g., urea, ammonium nitrate). Further research is required to understand mechanistic differences between amendment types. Potential increases in outbreak‐related herbivore deposits coupled with climate warming will accelerate litter decomposition and nutrient cycling rates with short‐term consequences for nutrient cycling and carbon storage in tropical montane wet forests.

## Graphical abstract

Our study examines the interactive effects of insect deposits (frass + cadavers) and mean annual temperature on litter decomposition along a highly constrained native‐dominated tropical montane wet forest in Hawaiʻi. We found that additional inputs of frass + cadavers immobilized nutrients as well as stimulated leaf litter decomposition, operating additively with increasing mean annual temperature. An understanding of insect responses tomultiple and potentially interacting natural and anthropogenic changes is necessary to improve our understanding of ecosystem responses to environmental change.

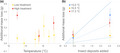

## 1 INTRODUCTION

Litter decomposition is a central process regulating carbon (C) and nutrient cycling within ecosystems, with impacts on both plant productivity and species composition (Knorr et al., 2005; Prescott, 2002). Understanding the mechanisms that control litter production and decomposition is, therefore, important to efforts seeking to accurately model C and N cycling. Litter quality strongly influences decomposition rates (Prescott, 2002), particularly in the initial phases of the decomposition process (Melillo et al., 1982). Furthermore, a global study of 70 published studies determined that C:N and total nutrient concentration (the sum of N, phosphorus, potassium, calcium, and magnesium concentrations) of litter explained 70.2% of the variability in litter decomposition rates, while MAT explained an additional 7.9% of the variability (Zhang et al., 2008). Theoretically, litter C:N should regulate net N release rates during litter decomposition because microbial decomposers release N only after their own N requirements have been met (Berg, 1986). Ultimately, patterns in net N release and immobilization may depend on the C:N of the decomposer organisms relative to that of the substrate, as well as N availability in the environment (Parton et al., 2007; and references therein).

Along with litter quality, climate has also long been shown to be a major driver of litter decomposition (Knorr et al., 2005, and references therein). Temperature modulates the rate at which microbial communities decompose leaf litter and mineralize nutrients through direct effects on their activity and indirect effects on community composition (García‐Palacios et al., 2013; Strickland et al., 2015; Wall et al., 2008). Many studies show that litter decomposition rates increase with rising temperatures, with the proportional change per every 10°C increase in temperature being described by *Q*_10_. Across studies, *Q*_10_ typically ranges from 2 to 3, with an additional variation often ascribed to the variation in litter quality, moisture availability, and microbial community composition (García‐Palacios et al., 2013). Exogenous N may stimulate chemical reactions between polyphenols and amino compounds, forming recalcitrant chemicals with a higher activation energy that subsequently slows decomposition (Fog, 1988). In this context, the temperature sensitivity of litter decomposition should increase as the net activation energy required for decomposition increases (Fierer et al., 2005). Slowing of microbial activity at cooler temperatures may inhibit decomposition even further. However, most temperature effect studies have been single‐factor investigations (Bothwell et al., 2014; Knorr et al., 2005), while multi‐factor studies examining interactions between temperature and other variables are less common (Salinas et al., 2011). One important set of understudied interactions involves climate‐mediated herbivore abundance and activity effects on litter decomposition (Kristensen et al., 2018).

Climate change and insect folivory can have significant consequences on soil processes in forests. Empirical studies and models predict insect outbreaks will intensify in response to climate change and other anthropogenic disturbances, including in tropical forests (Anderegg et al., 2015; Dyer et al., 2012; McDowell et al., 2018). While climate can have direct effects on insect performance (Bale et al., 2002), rising temperatures can have highly variable indirect effects on background levels of insect herbivory, reducing consumption in some studies while increasing consumption in others (Lemoine et al., 2014, and references therein). Such changes in ambient and outbreak levels of insect abundance and herbivory will affect the amount of foliar material consumed by insects and so production of frass, cadavers, leachate, and greenfall (Schowalter et al., 2011). Through these pathways, even background levels of herbivory by invertebrates can release as much or more of some nutrients as other major sources, with the potential to alter soil processes (Metcalfe et al., 2014). For example, frass can boost microbial growth (Frost & Hunter, 2004), thereby increasing litter decomposition rates (Zimmer & Topp, 2002), N mineralization rates, and N immobilization rates (Frost & Hunter, 2007; Lovett & Ruesink, 1995). Furthermore, relatively high concentrations of N and phosphorus (P) can be found in insect cadavers compared with frass (Kos et al., 2017; Ohmart et al., 1983). Given that the effect of invertebrate cadavers on plant growth and densities of aboveground and belowground organisms can be stronger than their living counterparts, the inclusion of insect cadavers in ecosystem process studies is important for understanding ecosystem level impacts (Kos et al., 2017).

Initial efforts to study temperature effects on herbivory rates have been species‐ and community‐specific (Lemoine et al., 2014, and references therein). Comparatively less is known about how insect herbivores collectively affect ecosystem‐level processes (Lindquist et al., 2012, but see Frost & Hunter, 2004). Preliminary work primarily in temperate regions provides compelling insights into the effects of insect herbivores and climate change on ecosystems such as reducing primary productivity and exacerbating invasive species problems (Couture et al., 2015; Ward & Masters, 2007). Multi‐factor in situ research is now needed to understand how insect herbivore frass and cadavers impact ecosystem processes in the understudied tropics. To address this gap, our study examined the effects of insect deposits (frass + cadavers) on litter decomposition rates across a highly constrained MAT gradient within a native‐dominated tropical montane wet forest in Hawaiʻi. To the best of our knowledge, this is the first study to investigate the effects of paired insect frass plus cadavers on litter decomposition across a temperature gradient. We used seven of nine previously established research plots spanning a 4.3°C MAT, 666‐m elevation gradient (Giardina et al., 2014; Litton et al., 2020). Instead of manipulating the ambient temperature of entire forest stands, an elevation gradient can provide a more tractable approach to assess the equilibrated response of entire, intact systems to climate change (Moser et al., 2011). While multiple environmental variables often co‐vary with temperature and elevation (Wood et al., 2012), the MAT gradient used in this study is ideally suited for asking questions about the effects of long‐term, whole‐forest temperature differences on ecosystem processes because potential drivers of ecosystem metabolism other than temperature (i.e., geological substrate, soil type and age, disturbance history, vegetation composition, soil moisture, and solar radiation) are nearly constant across elevation (Giardina et al., 2014; Litton et al., 2011; Selmants et al., 2016).

Within these seven MAT gradient plots, we examined for one year the decay of *Metrosideros polymorpha* leaf litter, a tree species that dominates native forests in this study system and across much of Hawaiʻi. Litterbags represent sets of three treatments were deployed in each of the seven plots, with three levels of insect deposits (control, low and high amounts of frass + cadavers). Our goal was to investigate how rates of leaf litter decomposition and N release from decomposing litter in a tropical montane wet forest may vary with rising temperature and additions of insect deposits.

According to a meta‐analysis by Knorr et al. (2005) across several ecosystem types, N additions inhibited litter decomposition when fertilization rates were 2–20 times greater than the anthropogenic level of N deposition, when ambient N deposition was 5–10 kg ha^−1^ yr^−1^, or when litter contained high levels of lignin. In contrast, decomposition rates increased at field sites exposed to low ambient N deposition (<5 kg ha^−1^ yr^−1^) and for low‐lignin litters. Based on this meta‐analysis, we hypothesized that while higher MAT would stimulate litter decomposition (Bothwell et al., 2014; Salinas et al., 2011), higher amendments of insect deposits would inhibit litter decomposition (Knorr et al., 2005). Specifically, we expected that litter decomposition would slow at our site where ambient levels of N deposition are high at 17 kg ha^−1^ yr^−1^ (Carrillo et al., 2002), additions are at least 20 times the ambient level of N deposition, and litter quality is low to medium (Hobbie & Vitousek, 2000). Further, we expected that the decelerating effect of insect deposits on litter decomposition would be greater for high versus low amendment amounts. Finally, we hypothesized that N mineralization would increase with temperature (Bothwell et al., 2014; Salinas et al., 2011), while C:N of remaining plant material would remain stable for the unamended, control litter but would decrease for the amended litters. Given the strong influence of temperature on litter decomposition rates (Coûteaux et al., 1995), we expected MAT to interact with exogenous N availability to significantly influence litter decomposition rates. A significant MAT × amendment interaction might result in significant differences in temperature sensitivity of litter decomposition between amendments.

## 2 STUDY SITE

We examined the effects of MAT and insect deposits on leaf litter decomposition along a highly constrained MAT gradient on the windward side of Maunakea Volcano, Hawaiʻi (Giardina et al., 2014; Litton et al., 2011, 2020). Airborne light detection and ranging measurements of forest structure and intensive ground‐based survey techniques were used to select plots representing maximum aboveground biomass at a given elevation while maintaining dominant tree species, geology, soil type, soil moisture, and disturbance history constant (Table 1). Plots were considered centered within larger stands of similarly sized forest. Typical on the windward side of Hawaiʻi Island, all plots along the gradient are classified as aggrading *M. polymorpha* – *Acacia koa* forests (Asner et al., 2009; Litton et al., 2020). Together, high‐canopy *M. polymorpha* and mid‐canopy *Cheirodendron trigynum* tree species comprise 84–97% of tree basal area across the plots (Litton et al., 2011; Selmants et al., 2014). Soils across the gradient a fall under the tephra‐derived, moderate to well‐drained hydrous, ferrihydritic/amorphic, isothermic/isomesic Acrudoxic Hydrudands classification (Giardina et al., 2014). The Akaka, Honokaa, Maile, and Piihonua soil series and soil age of ~20,000 years found across all plots are moderately common for Hawaii Island (Litton et al., 2020). Furthermore, soil pH, bulk density, base saturation, cation exchange, and soil water content are relatively constant across the plots (Litton et al., 2020; Lyu et al., 2021). Although mean annual rainfall changes across the MAT gradient (Table 1), mean monthly soil water content is nearly identical at all plots because declining rainfall is balanced by decreasing evapotranspiration driven by lower air temperatures with increasing elevation (Selmants et al., 2016). See Litton et al. (2011) for more site selection details. As a subset of the full MAT gradient, the seven plots used in this study span from 934 to 1600 m in elevation (13.0–17.3°C), with plots at the low end of the gradient in the Laupāhoehoe Unit of the Hawaiʻi Experimental Tropical Forest (19°56′15.3″N, 155°16′02.6″W) and at the high end in the Hakalau Forest National Wildlife Refuge (19°50′31.3″N, 155°17′35.2″W). The 4.3°C temperature change falls within the long‐term projected change for these ecosystems under the RCP‐8.5 scenario (Collins et al., 2013), and is close to the 4.4°C change projected for 2081–2100 under the SSPS‐8.5 scenario described in the IPCC 2021 summary report (Masson‐Delmotte et al., 2021). All seven permanent 20 m × 20 m plots are within 11 km of each other. Insect outbreaks are known to occur periodically in the Hawaiian Islands, which can result in massive defoliation events with subsequent extensive N deposition (Banko et al., 2016).

**TABLE 1:** Site characteristics across a 4.3°C mean annual temperature gradient in closed‐canopy tropical montane wet forests on the Island of Hawaiʻi.

Elevation (m)	Air temp. (°C)^a^	Rainfall (mm yr^−1^)^b^	Potential evapotranspiration (mm yr^−1^)^c^	Soil temp. (°C)^a^	Soil moisture (m^3^ m^−3^)^a^	Solar radiation (W m^−2^)^c^
934	17.3	4292	2232	17.3	0.55	200.9
1024	16.7	3975	2214	16.3	0.57	202.4
1116	16.1	3433	2137	15.6	0.51	210.1
1204	15.5	3181	2211	15.5	0.40	214.5
1274	15.1	3101	2234	14.9	0.51	216.2
1468	13.8	4119	1888	13.6	0.55	202.6
1600	13.0	3282	1961	12.6	0.57	213.1

^a^ Litton et al. (2011).

^b^ Giambelluca et al. (2013).

^c^ Giambelluca et al. (2014).

### 2.1 Experimental design

To minimize variation in litter nutrient concentrations, we used well‐mixed litter composed entirely of *M. polymorpha* leaves collected monthly from litterfall trays installed at the mid‐elevation (1116 m) between 2010 and 2014 along the full MAT gradient (see Giardina et al., 2014). Litter was oven‐dried (70°C), sorted, and stored in double‐sealed bags. To generate the large amount of insect frass and cadavers required for this study, spongy moths (*Lymantria dispar*) were raised on a standard artificial diet at the Otis Laboratory, United States Department of Agriculture in Buzzards Bay, Massachusetts to produce dried and sterilized frass + cadaver treatment mixtures. Total N of *L. dispar* frass at the start of the experiment was 65.80 mg g^−1^ with a C:N of 6.29. Total N of *L. dispar* cadavers was 151.00 mg g^−1^ with a C:N of 3.32. Initial N concentration for our standardized *M. polymorpha* leaf litter was 9.20 ± 0.19 mg g^−1^ with a C:N of 53.27.

We used a synthesis of insect energetics data (Wiegert & Petersen, 1983) to estimate two levels of frass + cadaver additions. Specifically, we assumed that 66% and 13% of insect ingested C would be transferred to frass and cadavers, respectively, resulting in our amendments being administered in a 5:1 ratio of insect frass to cadavers. Amendment levels represented ambient (Control), moderate (Amended‐Low), and severe (Amended‐High) natural insect outbreak events. We calculated the Amended‐Low amount (331 kg^−1^ ha^−1^) using data for actual litterfall rates of 1000 g m^−2^ y^−1^ of live foliage at our 1116 m plot (Litton et al., 2020) with the 34.6% foliage consumption rate being twice the background level of herbivory predicted for the sites (latitude ±1°, Zhang et al., 2016). We based the Amended‐High application (1818 kg^−1^ ha^−1^) on what might be expected during a major outbreak (100% foliage consumed), while relying on the same assumptions about insect energetics.

We constructed litterbags (11 cm × 11 cm) out of 1.0 mm × 1.5 mm mesh fiberglass screen filled with 2.5 g of well‐mixed senescent *M. polymorpha* leaves. Each of the seven MAT plots received 35 Control treatment litterbags (no amendments) and 35 litterbags receiving the Amendment‐Low treatment (low level additions of insect frass + bodies). Due to limited resources, only a subset of plots (at elevations 934, 1204, and 1600 m) received another set of 35 litterbags treated with an Amendment‐High treatment. We placed a cluster of five litterbags (one litterbag per collection period) with each of the two or three treatments tethered to a central stake in seven 5 m × 5 m subplots within each MAT plot, resulting in seven replicates for each treatment, collection period, and plot. Litterbags were placed at least 20 cm apart from one another.

We followed the decomposition rates of leaf litter in the seven MAT plots between March 2020 and March 2021, by collecting one litterbag of each treatment from each subplot at approximately 1, 3, 6, 9, and 12 months post‐deployment. After each collection, we carefully brushed off any debris from litter (including any insect deposits), then oven‐dried at 70°C, weighed, and finely ground litter for chemical analysis. We determined total C and N of initial, undecomposed leaf litter and of decomposed litter from the five collection periods by combustion using a Costech Elemental Analyzer at Lund University, Sweden (Costech Analytical Technologies; Valencia, CA USA).

### 2.2 Data analysis

We performed all statistical analyses in the R 4.2.1 statistical environment. Using the lme4 package (Bates et al., 2015) and lmer Test package (Kuznetsova et al., 2017), we generated linear mixed models to perform hypothesis testing using type III ANOVA and Tukey HSD analyses. We used amendment treatment (fixed) and MAT (random) as factors to determine whether leaf litter decomposition rate (*k*) per year varied significantly as a function of MAT and amendment level (*n* = 7, Appendix A) after approximately 12 months. Steps were taken to check that normality assumptions were met before proceeding with analyses. For each treatment, we estimated *k* rates for the seven replicate litterbag groups within each MAT plot by fitting a single exponential decay function to the litter mass data (Olson, 2007) using nonlinear regression with initial mass fixed at the initial value of 2.5 g (Adair et al., 2010):

(1) $$M_{t}=M_{0}\;e^{\mathrm{kt}}$$

where *M*_*t*_ is mass remaining at time *t* (in days) and *M*_0_ is initial mass. We fit the exponential decay function to the time series of litter mass loss using Excel Solver, by freely varying *k* until the root‐mean‐square error (RMSE) between predicted and observed litter mass over the five sampling intervals for each treatment and subplot was minimized (Appendix B). This procedure is identical to any commercial curve fitting software, but allowed us to explicitly specify the metric of fit between observed and predicted values and generate a single estimate of fit for all the data and not just the individual curves.

We calculated the temperature coefficient (*Q*_10_) for rates of litter decomposition across the MAT gradient as

(2) $$Q_{10}={\left(R_{2}/R_{1}\right)}^{10/\left(T_{2}-T_{1}\right)}$$

where *Q*_10_ is the proportional change in *k* due to a 10°C increase in MAT, *R*_1_ and *R*_2_ are regression‐derived estimates of *k* at the lowest and highest MAT plots, and *T*_1_ and *T*_2_ are MAT values of the lowest (13°C) and highest (17.3°C) MAT plots.

To estimate the proportion of initial N remaining, we divided the mass of N in litter by the mass of N in the initial undecomposed litter.

## 3 RESULTS

### 3.1 Effects of MAT and insect deposit amendments on litter decomposition

**FIGURE 1:**
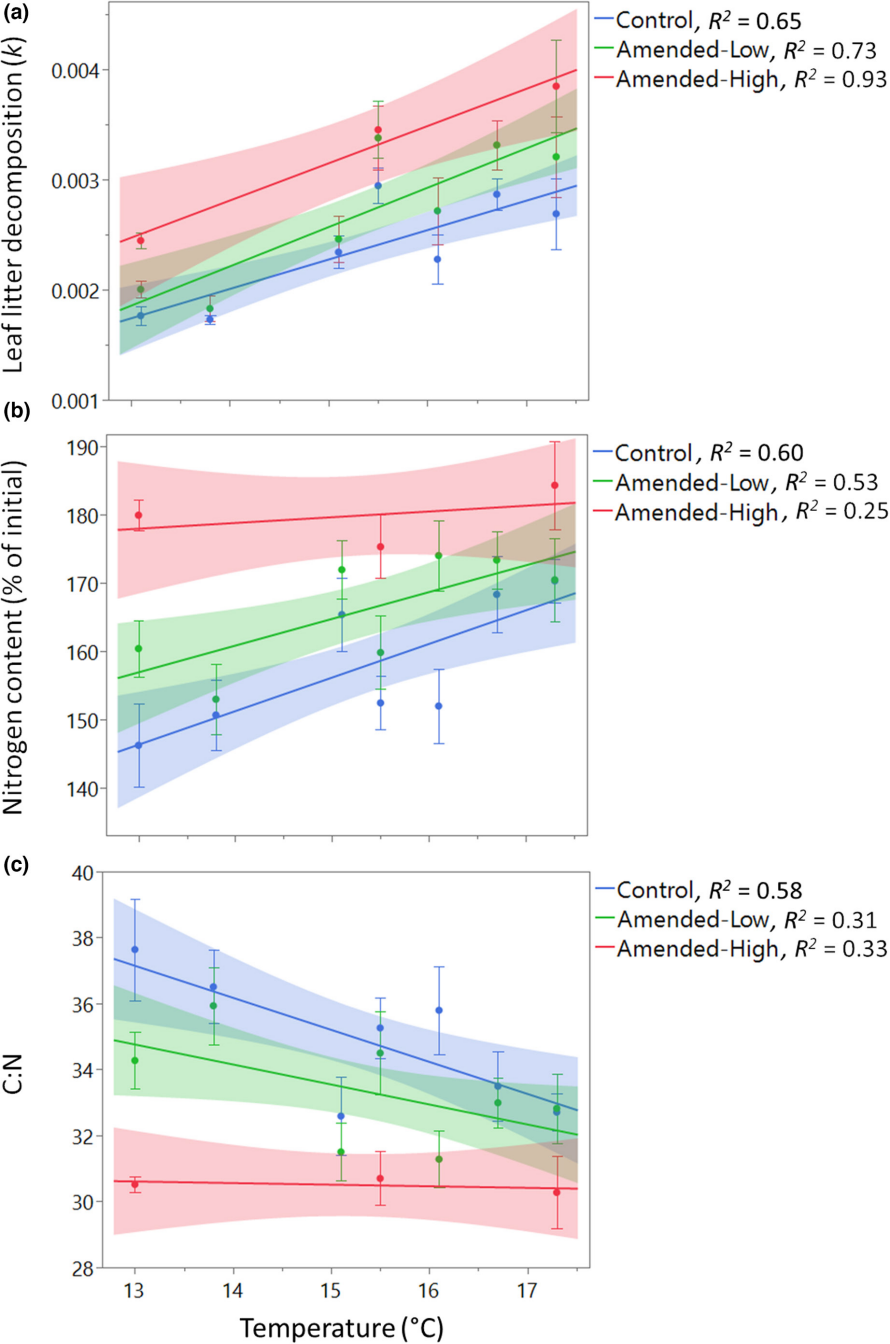
Insect frass + cadaver treatment and mean annual temperature (MAT) effects on: (a) litter decomposition rate (*k*), (b) N content, and (c) C:N across a 4.3°C MAT gradient after approximately 12 months of decomposition. Solid shapes denote means; bars denote ±1 *SE*, *n* = 7. Dashed lines denote best‐fitted lines and bands represent 95% confidence intervals.

For both litter Amendment and Control treatments, *k* increased with rising MAT (Figure 1a). Control litter *k* ranged from 1.73 × 10^−3^ d^−1^ to 2.94 × 10^−3^ d^−1^ across the MAT gradient and *k* was a positive function of MAT (*R* ^2^ = .65, F = 21.522, Ndf = 1, Ddf = 47, *p* < .001). Amended‐Low *k* ranged from 2.45 × 10^−3^ d^−1^ to 3.85 × 10^−3^ d^−1^ across MAT, with *k* being a positive function of MAT (*R* ^2^ = .73, F = 25.851, Ndf = 1, Ddf = 47, *p* < .008). And *k* for the Amended‐High treatment ranged from 1.83 × 10^−3^ d^−1^ to 3.38 × 10^−3^ d^−1^ across MAT, with *k* again being a positive function of MAT (*R* ^2^ = .93, F = 12.771, Ndf = 1, Ddf = 19, *p* < .001). Differences between treatments on litter mass remaining (averaged across MAT plots) over time can be found in Appendix C.

**FIGURE 2:**
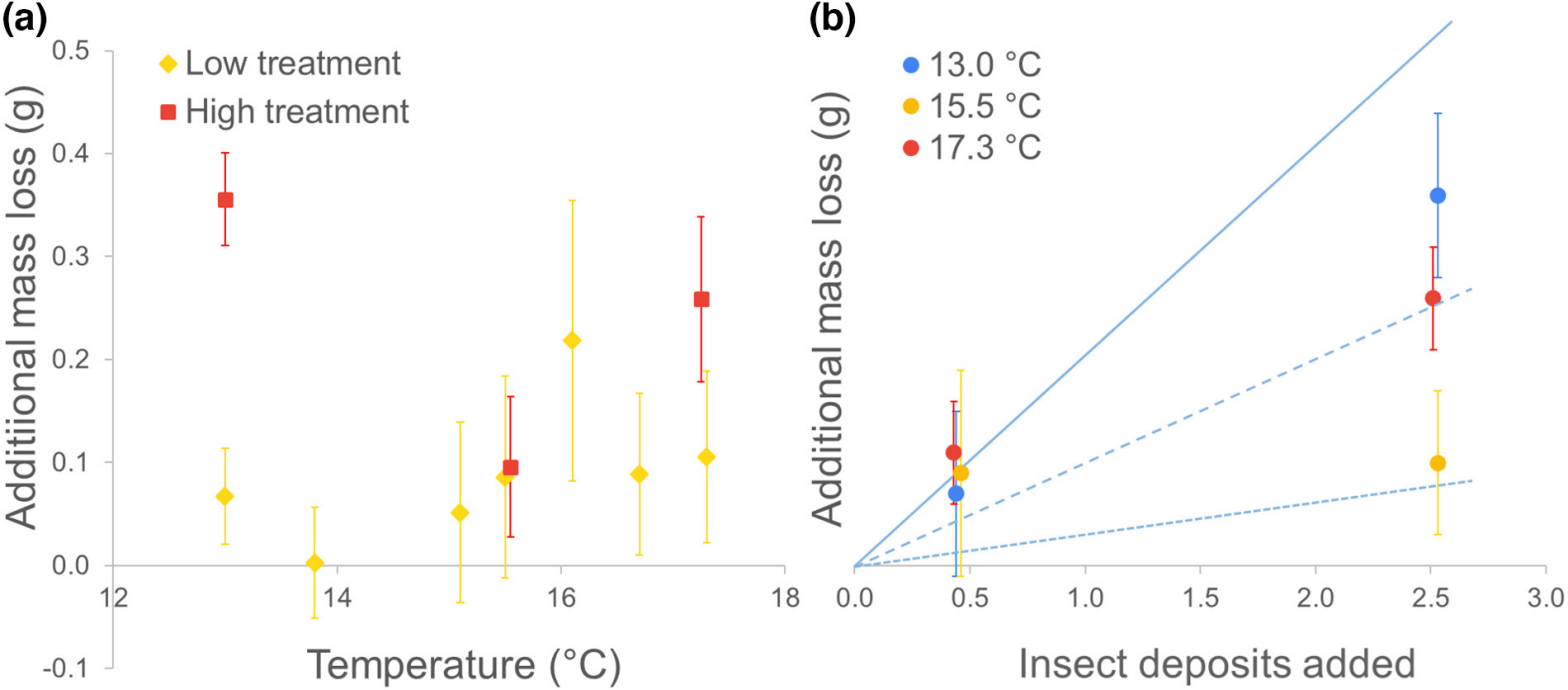
The (a) variability and (b) proportionality of additional leaf litter mass loss relative to control treatment due to two levels of insect frass + cadavers addition after 12 months of decomposition along a 4.3°C mean annual temperature gradient. Solid shapes denote means, and error bars denote ±1 *SE*, *n* = 7. Solid, dashed and dotted lines in panel b denote the 5:1, 10:1, and 30:1 lines for insect frass + cadavers mass added: Additional litter mass loss.

Decomposition rates increased additively with higher MAT and Amendment treatments with no interaction between Amendment treatments and MAT. Significant differences in *k* values were detected among treatments (*F* = 15.361, Ndf = 2, Ddf = 102, *p* = .003) and MAT (*F* = 14.765, Ndf = 6, Ddf = 102, *p* < .001), but not for treatment × MAT interactions (*F* = 0.503, Ndf = 8, Ddf = 102, *p* = .852). Specifically, after nearly 12 months of incubation (Figure 1a), the Amended‐High litter lost 15.9% more litter mass than Control litter (*F* = 6.569, Ndf = 3, Ddf = 36, *p* = .001) and 11.4% more litter mass than Amended‐Low litter (*F* = 6.569, Ndf = 3, Ddf = 36, *p* = .058) averaged across the lowest, middle, and highest MATs (Appendix D). Amended‐Low litter, however, had lost only 6.8% more mass than Control litter after nearly 12 months averaged across MAT (*F* = 6.569, Ndf = 7, Ddf = 84, *p* = .220, Figure 2a). Yet, Amended‐Low litter underwent larger cumulative litter mass loss at higher MATs, whereas mass loss for Amended‐High litter was more variable across MAT (Figure 2a).

We found that additional decomposition caused by the Amended‐High treatment was not proportional to the amount of additional insect deposit. That is, the Amended‐High treatment received five times the amount of frass + cadaver than the Amended‐Low treatment but additional decomposition in the Amended‐High treatment was less than four‐fold higher than Amended‐Low decomposition (Figure 2b).

### 3.2 *Q*_10_ of litter decomposition

Temperature sensitivity of litter was variable across treatments. The estimated *Q*_10_ values of *k* (mean ± *SE*) did not differ across the three treatments; *Q*_10_ was 2.66 ± 0.45, 2.99 ± 0.45, and 2.87 ± 0.41 for Control, Amended‐Low, and Amended‐High litter, respectively. Decomposition rates for Control, Amended‐Low, and Amended‐High treated litter increased significantly by 0.22 × 10^−3^ d^−1^, 0.28 × 10^−3^ d^−1^, and 0.33 × 10^−3^ d^−1^ for each 1°C increase in MAT, respectively (Figure 1a).

### 3.3 Nitrogen dynamics in decomposing litter

Nitrogen was immobilized in litter across MAT in all Amendment treatments. Litter amended with insect deposits had accumulated significantly more N than Control litter by the end of the year (*F* = 20.759, Ndf = 2, Ddf = 102, *p* < .001), where Amended‐High litter amassed the most N after 1 year (Figure 1b). The degree of decomposition‐associated N accumulation significantly increased with rising MAT (Figure 1b, *F* = 5.423, Ndf = 2, Ddf = 102, *p* < .001), with no treatment × MAT interaction (*F* = 1.138, Ndf = 8, Ddf = 102, *p* = .345).

The C:N of litter amended at both levels was lower than control litter overall but did not decline with MAT at the High‐Amended level. After 12 months, the C:N of Amended‐High litter was significantly lower than Amended‐Low litter (*F* = 19.089, Ndf = 3, Ddf = 36, *p* < .001). The C:N for both Amended‐Low (*F* = 19.089, Ndf = 7, Ddf = 84, *p* = .017) and Amended‐High (*F* = 19.089, Ndf = 3, Ddf = 36, *p* < .001) litter were significantly lower than C:N of the Control treatment. However, Amended‐High litter C:N was relatively constant across the MAT gradient (Figure 1c). The effect of the Amendment treatments on litter C:N did not interact with MAT after twelve months of decomposition (*F* = 1.402, Ndf = 8, Ddf = 102, *p* = .205).

## 4 DISCUSSION

Our elevation gradient provided a near‐ideal design for investigating temperature‐mediated effects on ecosystem processes because geological substrate, soil type and age, disturbance history, vegetation composition, soil moisture, and solar radiation were nearly constant across sites, allowing us to focus on rising temperatures. The consistency of these characteristics across plots is invaluable as in situ variation would confound study results. In line with prior studies across these sties (Bothwell et al., 2014; Giardina et al., 2014), we found that rising MAT stimulated litter decomposition rates (Figure 1a), which also aligned with prior findings of MAT‐driven increases in soil‐surface CO_2_ efflux and litterfall along this gradient (Giardina et al., 2014; Litton et al., 2011, 2020). We found that insect deposits had little apparent effect on *Q*_10_ values for litter decomposition, with the lack of a significant increase in the temperature sensitivity of *M. polymorpha* litter decomposition. Our results suggest that the litter microbial community along the gradient has a wide thermal tolerance and are insensitive to amendments.

Our hypothesis that additional insect deposits would inhibit litter decomposition (Knorr et al., 2005) was not supported. However, the Knorr et al. (2005) meta‐analysis did not examine the role of amendment type in litter decomposition across study systems. Hobbie and Vitousek (2000) conducted an amendment experiment in a nearby forest using similar litter as in our study, where they applied N chemically at 100 kg ha^−1^ yr^−1^, and found the additions had no significant effect on litter decomposition. They discussed that the demand for N may be low relative to supply because of the poor C quality of local litter, but also N fertilization may inhibit lignin degradation (see also Fog, 1988) and thereby offset stimulatory effects of N addition. Alternatively, some other factor may have constrained decomposition more strongly than N availability. That adding insect deposits accelerated litter decomposition in our study suggests that nutrient amendment as fertilizer versus insect frass + cadavers can lead to different stimulatory effects and that these differences may be site‐specific. Our study highlights that when nutrients are bound in organic forms, they can influence litter decomposition and nutrient release differently than when in inorganic fertilizer form, which is commonly used to study nutrient supplementation effects on ecosystem processes. Insect frass and cadavers can supply nutrients other than N, such as P, that also play a role in litter decomposition (Ohmart et al., 1983), and the passage of foliage through the gut of insects may alter nutrient quality or bacterial assemblages (Hammer et al., 2017; Lovett & Ruesink, 1995) that may then interact with litter, soil, and decomposers differently than do chemical fertilizers. A logical next step would be to conduct a fertilizer versus insect deposit study, where a comprehensive nutrient and microbial analysis of amendments and litter would be used to compare changes to litter decomposition processes.

Interestingly, the overall effects of MAT and our amendments were additive and not synergistic. Other studies on this gradient have demonstrated that rising MAT increases ecosystem C fluxes and soil nitrate availability (Giardina et al., 2014; Pierre et al., 2017), and alters the cycling, availability, and ecological stoichiometry of a suite of macro‐ and micronutrients (Litton et al., 2020). In agreement with the meta‐analysis by Knorr et al. (2005), the lack of a significant Amendment interaction in our study indicates that MAT may set boundaries for but not consistently alter the effect of N enrichment on litter decomposition.

In contrast to this study, other studies along our MAT gradient and in nearby *M. polymorpha* dominated forests have not observed N immobilization with increasing MAT (Bothwell et al., 2014) or MAT plus fertilization treatments (Hobbie & Vitousek, 2000). Greater rainfall during this study (Hawaii Permanent Plot Network, 2022) compared to the Bothwell et al.'s (2014) study may have made more nutrients available to microbes or altered microbial community composition (but see Selmants et al., 2016) and thus litter decomposition rates. In addition, it may be relevant that our litterbags were filled with more *M. polymorpha* leaf litter per area (0.23 g cm^−1^) than either the Bothwell et al.'s (2014) study (0.17 g cm^−1^) or the Hobbie and Vitousek's (2000) study (0.10–0.20 g cm^−1^). It is possible that the amount of leaf litter in our study crossed a threshold above which microbial activity and thus immobilization would be stimulated (Liu et al., 2017). Initial N concentration of leaf litter in our study (0.92% N) is low for senescent leaves of most forest species (Parton et al., 2007). At these levels, observed N immobilization aligns with findings from a global synthesis of litter chemistry and decomposition, and based on initial litter chemistry, our litter may not have decomposed sufficiently to begin releasing substantial amounts of N (Parton et al., 2007). This is line with other studies that have observed N immobilization during early stages of decomposition, sometimes followed by mineralization at later stages of decomposition (Zhou et al., 2018). Because the litter decomposition process is lengthy (Parton et al., 2007) and differences among treatments can be dynamic and complex (Chen et al., 2013; Melillo et al., 1982), a complete understanding of the fate of insect‐derived inputs requires longer tracer‐based experiments.

The influence of insect inputs on litter decomposition in our study may not be entirely explained by N availability and other factors such as limitation of other nutrients or decomposer community dynamics may co‐vary with N to influence litter decomposition (Hobbie, 2005). For example, differences in P supply and litter N:P can influence litter decomposition, microbial communities, and litter nutrient dynamics (Güsewell & Gessner, 2009). In fact, a recent study along this gradient demonstrated that MAT affects the cycling and availability of essential macro‐ and micronutrients, as well as the ecological stoichiometry of live foliage and litterfall (Litton et al., 2020). Ohmart et al. (1983) demonstrated that insect frass can be a source of not only N, but also P and potassium. The quality of insect frass can also affect soil nutrient availability, with effects spanning from positive to negative (Kagata & Ohgushi, 2012; Valdés‐Correcher et al., 2019). Such variation across MAT indicates that plot‐specific insect material may bring other factors to an already complex decomposition process. We therefore recommend future studies of frass and cadaver effects on nutrient dynamics use insect inputs from resident insect populations feeding on resident vegetation. Further, it would be valuable to study the circumstances under which nutrients from insect inputs are or are not mobilized by microbial activity or retained within recalcitrant organic matter. Separating the effects of frass from cadavers in future studies could also help refine our understanding of insect herbivore impacts on soil processes.

In contrast to a pattern of decreasing C:N for control and Amended‐Low treatments, C:N for Amended‐High litter was stable across the three low, medium, and high MAT plots in which the treatment was tested (Figure 1c). For Amended‐Low litter, the addition of exogenous N from insect deposits to litter may have stimulated microbes that were initially limited by N, leading to greater litter decomposition and litter N immobilization but lower C:N – especially in the first month of decomposition (Appendix E). That C:N was relatively constant across MAT for Amended‐High litter, suggests that C:N alone cannot explain accelerated rates of litter decomposition. When N is no longer limiting, MAT may have played a greater role in stimulating microbial activity and litter decomposition (Hobbie, 2005). Our results highlight the varying effect of MAT on litter decomposition processes under conditions of N limitation versus insect‐mediated enrichment, and underscore the complexity of predicting changes in soil processes under a changing climate.

Our study focused on the effects of insect deposits and MAT on surface leaf litter decomposition along a well‐constrained gradient for which confounding factors are largely controlled. However, because standing litter (litter that has not reached the soil surface) can contain more dissolved organic C than surface litter, the standing phase can play a critical role in litter decomposition and soil C storage (Wang et al., 2017). In one semi‐arid forest, standing litter decomposed faster than soil surface litter due to high dissolved organic C and leaf litter moisture content derived from relatively high night‐time humidity (Wang et al., 2017). Because of their importance especially in dry ecosystems, standing litter‐insect interactions also deserve attention in future multi‐factor studies.

This study highlights how insects and a warmer climate can combine to significantly impact ecosystem processes in tropical forests. Using a well‐constrained gradient, our results confirm that rising MAT increases litter decomposition rates in a tropical montane wet forest. Further, they demonstrate that herbivores can accelerate decomposition rates, and that leaf litter decomposition responses to insect frass and cadavers may operate additively to those of MAT. Finally, our results indicate that increased insect deposits immobilize nutrients in tropical wet montane forests and can operate differently from chemical fertilizers, warranting further investigation into the mechanisms responsible for observed differences among control and amendment types. An understanding of insect responses to multiple and potentially interacting natural and anthropogenic changes is necessary to improve our understanding of ecosystem responses to environmental change.

## AUTHOR CONTRIBUTIONS

**Bernice C. Hwang:** Conceptualization (lead); data curation (lead); formal analysis (lead); investigation (lead); methodology (lead); project administration (supporting); resources (supporting); software (lead); visualization (lead); writing – original draft (lead); writing – review and editing (lead). **Christian P. Giardina:** Funding acquisition (supporting); investigation (supporting); project administration (supporting); resources (supporting); supervision (supporting); validation (supporting); writing – review and editing (supporting). **Creighton M. Litton:** Funding acquisition (supporting); project administration (supporting); resources (supporting); writing – review and editing (supporting). **Kainana S. Francisco:** Data curation (supporting); investigation (supporting); project administration (supporting); supervision (supporting); writing – review and editing (supporting). **Cody Pacheco:** Data curation (supporting); investigation (supporting); project administration (supporting). **Naneaikealaula Thomas:** Data curation (supporting); investigation (supporting); project administration (supporting). **Tyler Uehara:** Data curation (supporting); investigation (supporting); project administration (supporting). **Daniel B. Metcalfe:** Conceptualization (supporting); formal analysis (supporting); funding acquisition (lead); investigation (supporting); project administration (supporting); resources (lead); supervision (supporting); writing – review and editing (supporting).

## CONFLICT OF INTEREST

The authors have no competing interests or conflict of interest to declare.

## Data Availability

Data are publicly available at https://doi.org/10.6084/m9.figshare.19085612.v1.

## APPENDIX A

ANOVA and Tukey HSD results for litter decomposition (*k*) using entire time series. Statistically significant changes (95%) are indicated in bold.

## APPENDIX B

Relation between litter mass observed and predicted with the exponential decay model fit the pattern of mass loss over five sampling intervals for each treatment and subplot. The solid black line indicates a 1:1 relationship; the dotted black line indicates a linear regression (*R* ^2^ = .91, RMSE = 0.17).

## APPENDIX C

Treatment effects on litter mass remaining averaged across all MAT plots over time. Solid shapes denote means; bars denote ±1 *SE*, *n* = 7. Solid lines denote best fitted lines.

## APPENDIX D

Treatment differences in leaf litter mass remaining a 4.3°C MAT gradient. Solid shapes denote means; bars denote ±1 *SE*, *n* = 7. Solid lines denote best‐fitted lines and bands represent 95% confidence intervals.

## APPENDIX E

ANOVA and Tukey HSD results for N remaining (% of initial) and C:N at each time collection. Significant differences (95% confidence level) indicated in bolded print.
